# Promoter Methylation of p16^INK4A^, hMLH1, and MGMT in Liquid-Based Cervical Cytology Samples Compared with Clinicopathological Findings and HPV Presence

**DOI:** 10.1155/2011/927861

**Published:** 2011-06-04

**Authors:** Aris Spathis, Evaggelia Aga, Maria Alepaki, Aikaterini Chranioti, Christos Meristoudis, Ioannis Panayiotides, Dimitrios Kassanos, Petros Karakitsos

**Affiliations:** ^1^Department of Cytopathology, University General Hospital “ATTIKON”, School of Medicine, Rimini 1, Chaidari, 12462 Athens, Greece; ^2^Department of Pathology, University General Hospital “ATTIKON”, School of Medicine, Rimini 1, Chaidari, 12462 Athens, Greece; ^3^3rd Department of Obstetrics and Gynecology, University General Hospital “ATTIKON”, School of Medicine, Rimini 1, Chaidari, 12462 Athens, Greece

## Abstract

Cervical cancer is a common cancer inflicting women worldwide. Even though, persistent infection with oncogenic Human Papillomavirus (HPV) types is considered the most important risk factor for cervical cancer development, less than 5% of women with HPV will eventually develop cervical cancer supporting that other molecular events, like methylation-dependent inactivation of tumor suppressor genes, may cocontribute in cervical carcinogenesis. We analyzed promoter methylation of three candidate genes (p16, MGMT, and hMLH1) in 403 liquid-based cytology samples. Methylation was commonly identified in both benign and pathologic samples and correlated with higher lesion grade determined by cytological, colposcopical, or histological findings, with HPV DNA and mRNA positivity of specific HPV types and p16^INK4A^ protein expression. Overall accuracy of methylation is much lower than traditional diagnostic tests ranking it as an ancillary technique with more data needed to identify the exact value of methylation status in cervical carcinogenesis.

## 1. Introduction

Cervical cancer still remains the third most commonly diagnosed cancer type in women worldwide, particularly in developing countries, with over 500,000 estimated new cases and over 250,000 estimated deaths [1]. The main cause of cervical cancer development is infection with Human Papilloma Viruses (HPVs) [2], that are small DNA viruses with oncogenic properties [3–5]. There are over 100 different HPV types, but only around 40 have been found in cervical epithelium and about 20 have been considered as high-risk factors for cancer development [6, 7]. Even though, persistent infection with oncogenic Human Papillomavirus (HPV) types is considered the most important risk factor for cervical cancer development, less than 5% of women with HPV will eventually develop cervical cancer [8], supporting the notion that other molecular events cocontribute in cervical carcinogenesis.

Inactivation of tumor suppressor genes has been shown to be a critical step in tumor development [9]. Apart from well-monitored suppression mechanisms as mutational inactivation, chromosome deletions, and loss of heterozygosity, epigenetic inactivation of tumor suppressor genes is a more recent discovery, where promoter methylation of a tumor suppressor gene abolishes its expression [10]. A significant amount of studies have provided evidence that promoter methylation of tumor suppressor genes is linked with cervical carcinogenesis [11–13] and even with specific severity of lesions [14].

Methylation-specific PCR (MSP) is a sensitive technique widely used to identify promoter methylation, mainly due to its low cost [15]. With MSP, promoter methylation has been discovered in various tumor suppressor genes connected with cell cycle regulation as p16^INK4A^ and DNA repair mechanisms as human MutL Homolog 1 (hMLH1) and O6-Methylguanine DNA Methyl Transferase (MGMT) [11, 13, 16, 17]. p16^INK4A^ is a protein shown to be overexpressed in high-grade lesions as a result of HPV oncoprotein over-expression, while inhibition of DNA repair mechanisms has been shown to occur in many types of carcinomas [4, 5, 9, 13]. 

In this study we used MSP to identify promoter methylation of the three above referred tumor suppressor genes in normal and pathological cervical liquid-based cytology samples, in order to evaluate their use in identifying lesions. Next we assessed the relation of promoter methylation to HPV presence, mRNA expression, p16^INK4A^ protein expression, and clinicopathological features, in order to clarify whether methylation is correlated with HPV presence and lesion progression.

## 2. Materials and Methods

### 2.1. Specimens

Samples were part of a larger pool of samples from primary screening for cervical cancer in Greece. A total of 403 liquid-based cytological (LBC) smears from women that underwent colposcopy were included in the present study. These consisted of 340 histologically confirmed samples and 63 samples with normal cytology that were added in order to increase the number of cytologically negative samples and have a better baseline of promoter methylation in “normal” samples. The study population consisted of women with a mean of 36.8 years of age (min–max: 18–81), a start of sexual intercourse at 18.9 years of age (13–30), and with a mean of 3.9 sexual partners (1–16). Cytology smears were collected in liquid-based media (ThinPrep, Hologic, Marlborough, USA), a single-layer smear was prepared by automated means (TP2000 processor), stained according to Papanikolaou, and diagnosis was set according to the Bethesda system by a skilled cytopathologist [18]. All molecular tests were performed on residual LBC specimens. Histology diagnosis was set by a skilled histopathologist and for statistical purposes CIN-I were classified as LSIL, while CINII and CINIII were classified as HSILs.

### 2.2. Bisulfite Conversion MSP

**Figure 1:**
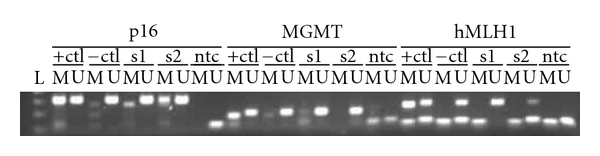
Agarose gel electrophoresis of PCR products of gene promoter methylation of (a) MGMT, (b) hMLH1, and (c) p16^INK4A^. L: DNA ladder 50 bp, +ctl: DNA treated with SssI, −ctl: unmethylated DNA control, s1,s2: clinical samples negative for methylation for MGMT and hMLH1. s2 is positive for p16 methylation.

A commercially available kit for bisulfate conversion and PCR amplification was used (Amplicolon, Bird Srl, Siena, Italy) to identify promoter methylation of p16^INK4A^, hMLH1, and MGMT. PCR products were analyzed in a 2% agarose gel, stained with ethidium bromide and visualized under UV light. If a PCR product was detected only in the unmethylated reaction, sample was characterized as unmethylated, while presence of a PCR product in the methylated reaction characterized the sample as methylated, regardless of the result of the unmethylated reaction. Absence of a product from both reactions characterized the sample as invalid. An unmethylated DNA control is included in the kit, while a methylated DNA control was created after treatment of the unmethylated control with SssI methyltransferase (NEB, Massachusetts, USA) (see Figure 1).

### 2.3. HPV Typing and E6/E7 mRNA Expression

DNA HPV typing was performed using a commercially available kit (CLART HPV2, Genomica, Madrid, Spain), according to manufacturer's instructions. The kit can identify 35 different HPV types, that are categorized as low risk (HPVs 6, 11, 40, 42, 43, 44, 54, 61, 62, 71, 72, 81, 83, 84, and 89) and high risk (HPVs 16, 18, 26, 31, 33, 35, 39, 45, 51, 52, 53, 56, 58, 59, 66, 68, 70, 73, 82, and 85) based on their epidemiology in specific grade of lesions [6]. E6/E7 mRNA expression was identified using the commercially available Nuclisens EasyQ HPV kit (Biomerieux SA, Marcy l'Etoile, France), that is, able to detect mRNA of five high-risk HPV types (HPV16, HPV18, HPV31, HPV33, and HPV45). Positive and no template controls were included in all experiments.

### 2.4. p16^INK4A^ Protein Expression

Protein expression of p16^INK4A^ was identified by immunocytochemistry using a commercial kit according to the manufacturer's instructions (CINtec Cytology, mtm Laboratories AG, Heidelberg, Germany). Evaluation of positive staining was performed by an experienced cytopathologist.

### 2.5. Statistical Analysis

All statistic tests were performed using IBM Statistics SPSS 19 (IBM Corporation, NY, USA) using *χ* ^2^ analysis, two-paired *t*-test and bivariate correlation analysis. All analysis tests were two tailed with significance at 95%. Overall accuracy was estimated by ROC analysis using histology (HSIL+) as the golden standard.

## 3. Results and Discussion

Promoter methylation status was successfully analyzed for 403 samples for MGMT, 372 samples for hMLH1 and 290 samples for p16^INK4A^. p16^INK4A^ protein expression was available for 358 samples, HPV DNA typing for 398 samples and mRNA expression for 355 samples. Cytological, colposcopical, and histological findings were well correlated as indicated by the area under the curve (AUC) values of both cytology (0.863) and colposcopy (0.861) compared to histology.

### 3.1. Promoter Methylation and Clinicopathological Findings

Results of promoter methylation status are summarized in Table 1. MGMT methylation was the most aberrant methylated gene, followed by p16^INK4A^ and finally by hMLH1. MGMT methylation increased statistically significant with lesion severity as determined by either cytological (*P* < .0001), colposcopical (*P* < .0001), or histological (*P* < .0001) findings. hMLH1 methylation, on the other hand, displayed a significant increase with lesion severity only with cytological (*P* = .0173) and colposcopical (*P* = .0489) findings, while p16^INK4A^ methylation showed no significant difference. Any of the three genes was statistically more often methylated in more severe lesions, as determined by either cytological (*P* < .0001), colposcopical (*P* = .0002), or histological (*P* = .0031) findings.

### 3.2. Promoter Methylation and Molecular/Immunocytochemical Findings

Positivity of p16^INK4A^ protein expression increased with MGMT and p16^INK4A^ methylation (*P* = .001 and *P* = .013). Methylation of MGMT increased with HPV DNA positivity (*P* = .021), overall mRNA positivity (*P* = .017), expression of HPV16 mRNA (*P* = .001), and DNA positivity for HPV16, HPV18, and HPV68 (*P* < .01). hMLH1 methylation increased with positivity for low-risk HPV DNA (*P* = .001), HPV16 mRNA (*P* = .035) and DNA positivity for HPV40, HPV51, and HPV61 (*P* < 0.01). Methylation of p16^INK4A^ increased with overall mRNA positivity (*P* = .046), expression of HPV16 mRNA (*P* = .05), HPV33 mRNA (*P* = .036) HPV16, HPV43, and HPV85 DNA positivity. Presence of either HPV16, HPV45, HPV53, HPV61, HPV68 DNA positivity or HPV16 and HPV45 mRNA positivity was correlated with an increase of the number of methylated genes (*P* < .01).

### 3.3. HPV Status, p16^INK4A^ Protein Expression and Clinicopathological Findings

Severity of the lesion, whether determined by cytology, colposcopy, or histology, was statistically higher with p16^INK4A^ protein expression, mRNA expression, and presence of HPV DNA of high-risk types (*P* < .001). HPV DNA positivity could either be only from high-risk types or mixed with low-risk types. Presence of low-risk HPV DNA was only correlated with low grade lesions (*P* < .01). HPV DNA positivity was more common in younger women (35.5 versus 39.3, *P* = .003) with more sex partners (4.3 versus 2.8, *P* = .039), while p16^INK4A^ protein expression was more common in older women (39.6 versus 36.8, *P* = .04) with earlier sex life initiation (17.8 versus 19.5, *P* = .019).

### 3.4. Discussion

Promoter methylation has been proposed to be a significant cofactor in carcinogenesis, especially in nonhereditary carcinomas. Its role in epigenetic inactivation of tumor suppressor genes has been shown to be common in many types of carcinomas, while recent evidence supports its contribution in cancer development in the cervix [10–14]. 

Findings of this study, as far as promoter methylation increase during lesion progression is concerned, are partially consistent with previous studies [11, 12, 14, 19]. The main difference with the present study is the significantly larger amount of samples that are included in our study and the significantly higher positivity rate of methylation in our study. As far as diagnostic or screening utility of promoter methylation, none of the genes displayed an AUC of over 0.6 when plotted against histology, with HSIL as the cutoff, ranking it as an ancillary technique with more data needed to identify the exact value of methylation status in cervical carcinogenesis.

**Figure 2:**
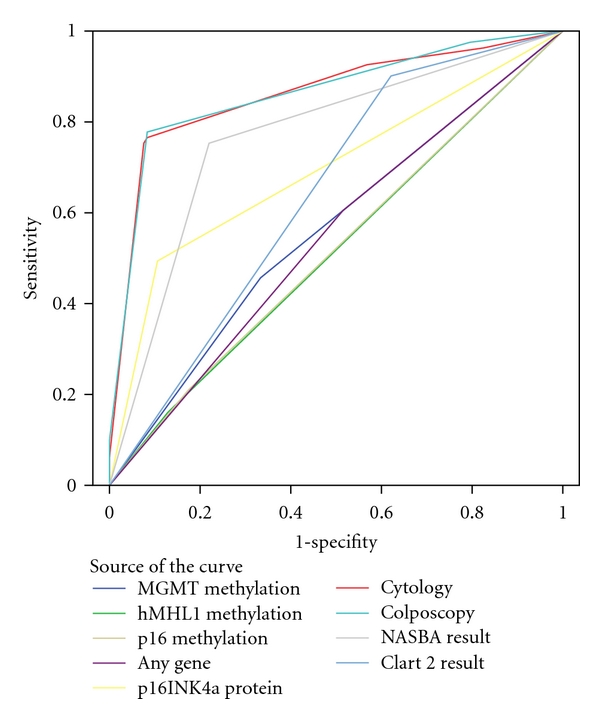
ROC curve analysis. Diagonal segments are produced by ties.

As expected HPV DNA testing displayed high sensitivity with low specificity depicted in the ROC curve (Figure 2, AUC 0.641), while mRNA higher specificity with lower sensitivity (AUC 0.767). p16^INK4A^ protein expression showed displayed better specificity than mRNA testing, but worse sensitivity (AUC 0.694). The above findings are consistent with previous studies, that have shown the use of these techniques in triaging women [6, 7, 20–23].

Presence of both p16^INK4A^ protein expression and promoter methylation was identified in both HSILs and carcinomas. Due to the heterogeneity of cytological samples this could reflect different pathways that are activated in specific types of cells or could be due to the partial methylation that can be identified with MSP but is not enough to abolish protein expression [24]. MGMT methylation was correlated with previously reported risk factors for severe lesions [7, 20, 21, 25], while interestingly enough hMLH1 methylation was correlated with low-risk HPV types especially when co-infection of a low-risk with a high-risk was identified.

## 4. Conclusion

Aberrant DNA promoter methylation of MGMT, hMLH1, and p16^INK4A^ is a common finding in liquid-based cytology samples of the cervix. Even though, there is a statistically significant increase of DNA methylation as the severity of the lesion increases, either for a single gene, or for the total number of methylated genes, the accuracy of promoter DNA methylation in identifying severe lesions is low. As a result wide use in screening programs is not recommended, since more studies with larger methylation panels should be performed before the exact significance of methylation in cervical carcinogenesis is elucidated.

## Figures and Tables

**Table 1:** Promoter methylation results.

		MGMT				hMLH1				p16^INK4A^				Any gene			
		*M*	(%)	*N*	*P*	*M*	(%)	*N*	*P*	*M*	(%)	*N*	*p*	*M*	(%)	*N*	*P*
Cytology	WNL	22	22.7	97	***	5	5.7	88	*	7	13.5	52		31	32.0	97	***
	ASCUS	26	40.7	64		4	7.0	57		9	18.4	49		33	51.6	64	
	LgSIL	51	38.6	132		22	17.9	123		18	17.5	103		75	56.8	132	
	ASC-H	3	37.5	8		1	14.3	7		1	33.3	3		4	50.0	8	
	HgSIL	43	47.3	91		13	14.8	88		15	19.7	76		54	59.3	91	
	SCC	5	80.0	6		0	0	5		1	33.3	3		5	83.3	6	
	AdenoCa	5	100	5		2	50.0	4		3	75.0	4		5	100	5	

Colposcopy	NSF	3	42.9	7	***	0	0	7	*	0	0	5		3	42.9	7	***
	Negative	25	27.2	93		6	7.3	82		11	21.2	52		35	38.0	93	
	LGSIL	74	38.7	191		24	13.6	177		25	17.6	142		100	52.4	191	
	HGSIL	40	40.8	98		15	16.1	93		15	18.5	81		56	57.1	98	
	SCC	9	81.8	11		1	10.0	10		1	14.3	7		9	81.8	11	
	AdenoCa	4	100	4		1	33.3	3		2	66.7	3		4	100	4	

Histology	Negative	13	22.8	57	***	8	15.1	53		5	12.2	41		21	36.8	57	**
	LSIL	70	42.7	164		21	13.5	155		25	20.7	121		95	57.9	164	
	HSIL	43	42.6	101		14	14.6	96		15	17.6	85		57	56.4	101	
	SCC	8	66.7	12		1	9.1	11		1	12.5	8		8	66.7	12	
	AdenoCa	6	100	6		2	40.0	5		3	75.0	4		6	100	6	

*M*: Methylated, *N*: Number of cases, NSF: Nonsatisfactory, *χ* ^2^ for trend *P*: *P < .05, **P < .005, ***P < .001.
